# Enrofloxacin hydro­chloride dihydrate

**DOI:** 10.1107/S1600536814006059

**Published:** 2014-03-26

**Authors:** Jorge E. Miranda-Calderón, Lilia Gutiérrez, Marcos Flores-Alamo, Ponciano García-Gutiérrez, Héctor Sumano

**Affiliations:** a Departamento de Fisiología y Farmacología, Facultad de Medicina Veterinaria y Zootecnia, Universidad Nacional Autónoma de México, Av. Universidad 3000, Delegación Coyoacán, Ciudad de México, CP 04510, Mexico; b Facultad de Química, Universidad Nacional Autónoma de México, 04510 México D.F., Mexico; c Laboratorio Divisional de Espectroscopia de Masas, Universidad Autónoma Metropolitana-Iztapalapa, Av. San Rafael Atlixco 186, Delegación Iztapalapa, Ciudad de México, CP 09340, Mexico

## Abstract

The asymmetric unit of the title compound, C_19_H_23_FN_3_O_3_^+^·Cl^−^·2H_2_O [systematic name: 4-(3-carb­oxy-1-cyclo­propyl-6-fluoro-4-oxo-1,4-di­hydro­quin­o­lin-7-yl)-1-ethyl­piperazin-1-ium chloride dihydrate], consists of two independent monocations of the protonated enrofloxacin, two chloride anions and four water mol­ecules. In the cations, the piperazinium rings adopt chair conformations and the dihedral angles between the cyclo­propyl ring and the 10-membered quinoline ring system are 56.55 (2) and 51.11 (2)°. An intra­molecular O—H⋯O hydrogen bond is observed in each cation. In the crystal, the components are connected *via* O—H⋯Cl, N—H⋯Cl and O—H⋯O hydrogen bonds, and a π–π inter­action between the benzene rings [centroid–centroid distance = 3.6726 (13) Å], resulting in a three-dimensional array.

## Related literature   

For the biological activity of enrofloxacin, see: Sárközy (2001 ▶); Sumano & Gutierrez (2013 ▶). For a related structure, see: Yamuna *et al.* (2014 ▶). For hydrogen-bond motifs, see: Etter *et al.* (1990 ▶). For standard bond-length data, see: Allen *et al.* (1987 ▶). For ring conformations, see: Cremer & Pople (1975 ▶); Duax *et al.* (1976 ▶).
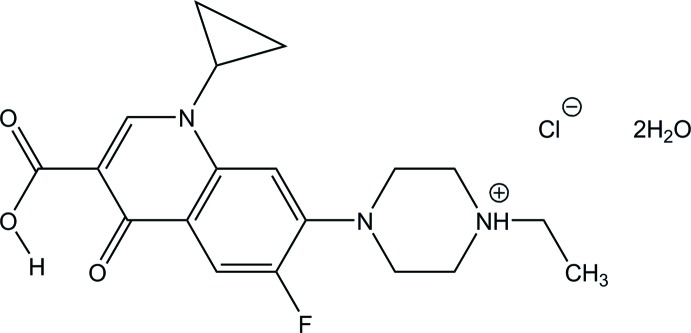


## Experimental   

### 

#### Crystal data  

C_19_H_23_FN_3_O_3_^+^·Cl^−^·2H_2_O
*M* *_r_* = 431.88
Monoclinic, 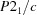

*a* = 7.1874 (3) Å
*b* = 21.1475 (8) Å
*c* = 26.5106 (10) Å
β = 91.407 (4)°
*V* = 4028.3 (3) Å^3^
*Z* = 8
Mo *K*α radiation
μ = 0.24 mm^−1^
*T* = 130 K
0.47 × 0.14 × 0.04 mm

#### Data collection  

Agilent Xcalibur (Atlas, Gemini) diffractometer
Absorption correction: analytical (*CrysAlis RED*; Agilent, 2012 ▶) *T*_min_ = 0.939, *T*_max_ = 0.992
18668 measured reflections
9291 independent reflections
5799 reflections with *I* > 2σ(*I*)
*R*_int_ = 0.044

#### Refinement  

*R*[*F* ^2^ > 2σ(*F* ^2^)] = 0.058
*wR*(*F* ^2^) = 0.132
*S* = 1.02
9291 reflections
561 parameters
12 restraints
H atoms treated by a mixture of independent and constrained refinement
Δρ_max_ = 0.39 e Å^−3^
Δρ_min_ = −0.29 e Å^−3^

### 

Data collection: *CrysAlis PRO* (Agilent, 2012 ▶); cell refinement: *CrysAlis PRO*; data reduction: *CrysAlis RED* (Agilent, 2012 ▶); program(s) used to solve structure: *SHELXS2013* (Sheldrick, 2008 ▶); program(s) used to refine structure: *SHELXL2013* (Sheldrick, 2008 ▶); molecular graphics: *Mercury* (Macrae *et al.*, 2006 ▶); software used to prepare material for publication: *WinGX* (Farrugia, 2012 ▶).

## Supplementary Material

Crystal structure: contains datablock(s) global, I. DOI: 10.1107/S1600536814006059/is5348sup1.cif

Structure factors: contains datablock(s) I. DOI: 10.1107/S1600536814006059/is5348Isup2.hkl

Supporting information file. DOI: 10.1107/S1600536814006059/is5348Isup3.cml

CCDC reference: 992455

Additional supporting information:  crystallographic information; 3D view; checkCIF report

## Figures and Tables

**Table 1:** Hydrogen-bond geometry (Å, °)

*D*—H⋯*A*	*D*—H	H⋯*A*	*D*⋯*A*	*D*—H⋯*A*
O3*W*—H3*D*⋯Cl1	0.888 (18)	2.27 (2)	3.126 (2)	163 (3)
O2*W*—H2*E*⋯Cl1	0.859 (17)	2.363 (19)	3.207 (2)	167 (3)
O4*W*—H4*E*⋯O3*A* ^i^	0.895 (18)	2.015 (19)	2.899 (3)	169 (3)
O3*W*—H3*E*⋯Cl2	0.883 (18)	2.52 (2)	3.356 (3)	158 (3)
O1*W*—H1*E*⋯Cl2	0.866 (18)	2.350 (18)	3.215 (2)	179 (3)
O4*W*—H4*D*⋯Cl2^ii^	0.875 (17)	2.325 (19)	3.190 (2)	170 (3)
O1*W*—H1*D*⋯Cl1	0.858 (18)	2.45 (2)	3.285 (2)	163 (3)
O2*W*—H2*D*⋯O3*B* ^iii^	0.886 (17)	1.940 (18)	2.819 (3)	172 (3)
O2*A*—H2*F*⋯O1*A*	0.876 (17)	1.68 (2)	2.523 (2)	160 (3)
O2*B*—H2*G*⋯O1*B*	0.871 (17)	1.73 (2)	2.532 (3)	152 (3)
N3*B*—H3*G*⋯Cl1	0.942 (16)	2.219 (17)	3.154 (2)	172 (2)
N3*A*—H3*F*⋯Cl2^iv^	0.946 (16)	2.204 (17)	3.149 (2)	177 (2)

Symmetry codes: (i) 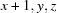; (ii) 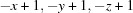; (iii) 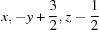; (iv) 
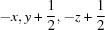
.

**Fig. 1.**
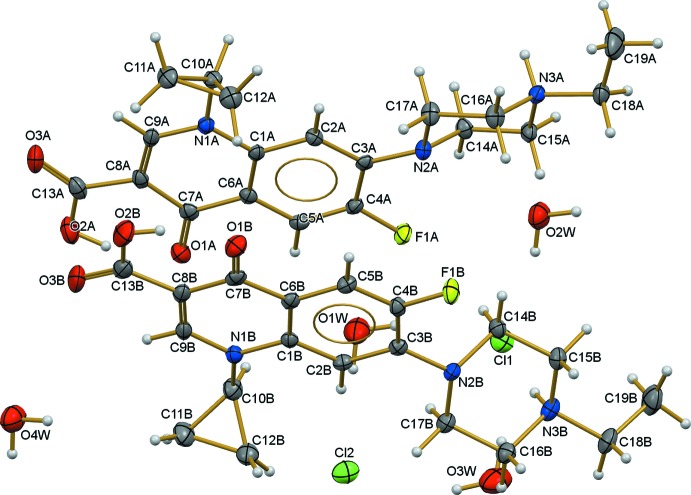
The molecular structure of the title compound. Displacement ellipsoids are drawn at the 50% probability level and H atoms are shown as circles of arbitrary size.

**Fig. 2.**
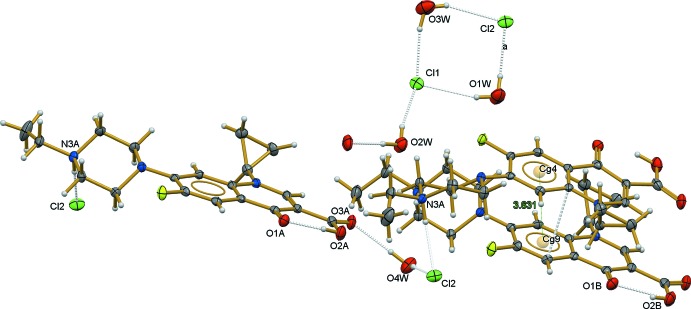
Intramolecular O—H···O hydrogen bonds, intermolecular hydrogen bonds forming the R42(8) and C32(17) motifs and a π–π interaction between A and B molecules.

## supplementary crystallographic information

### 1. Comment

Enrofloxacin is a synthetic antimicrobial agent that belongs to the group of synthetic 6-fluoroquinolones (Sárközy, 2001). Essential for the broad spectrum and the excellent antimicrobial efficacy is the fluorine substituent at position C6 and the piperazine ring at C7. The development of pharmaceutical derivatives of active principles such as salts, represent extensions of chemical space wherein enhanced or new chemical and physical properties may lead to extended use of a given drug as a therapeutic agent. Hence, the aim of this trial was produce enrofloxacin hydrochloride salt in order to improve its pharmacokinetic behavior (Sumano & Gutierrez, 2013).

The title compound crystallizes with two independent monocations (*A* and *B*) of the enrofloxacin protonated (*EnrH^+^*), two chloride anions and four water molecules solvent (Fig. 1). The piperazinium rings in both the cations adopt chair conformations. For *A* molecule, puckering parameters (Cremer & Pople, 1975) are Q = 0.588 (3) Å, θ = 178.42 (19), φ = 346 (12)° (if the calculation starts from N2A to C17A and proceeds in a counterclockwise direction) with asymmetry parameters (Duax *et al.*, 1976): ΔC_2_(N2A—C14A) = 0.8 (3), ΔC_2_(C14A—C15A) = 0.6 (3), ΔC_2_(C15A—N3A) = 0.4 (3), ΔC_s_(N2A) = 0.6 (2), ΔC_s_(C14A) = 0.2 (2), ΔC_s_(C15A) = 0.7 (2) and ΔC_s_(C17A) = 0.7 (2)° with a weighted average absolute torsion angle of 58.20 (12)° and weighted average ring bond distance of 1.4931 (13) Å. For *B* molecule, puckering parameters are Q = 0.586 (3) Å, θ = 3.8 (2), φ= 200 (4)° (if the calculation starts from N2B to C17B and proceeds in a counterclockwise direction) with asymmetry parameters: ΔC_2_(N2B—C14B) = 0.4 (3), ΔC_2_(C14B—C15B) = 3.3 (3), ΔC_2_(C15B—N3B) = 3.5 (3), ΔC_s_(N2B) = 1.9 (2), ΔC_s_(C14B) = 1.4 (2), ΔC_s_(C15B) = 3.2 (2) and ΔC_s_(C17B) = 3.2 (2)° with a weighted average absolute torsion angle of 57.93 (12)° and weighted average ring bond distance of 1.4930 (13) Å. Bond lengths are in normal ranges (Allen *et al.*, 1987) and to previously reported (Yamuna *et al.*, 2014). The dihedral angles between the mean planes of the cyclopropyl ring and the 10-membered quinoline ring are 56.55 (2) and 51.11 (2)° for *A* and *B* molecules, respectively.

In each cation, an intramolecular O—H···O hydrogen bond is observed [O2A—H2F···O1A and O2B—H2G···O1B (Table 1 & Fig. 2)]. In the crystal, there are classic hydrogen bonds (Table 1) mainly between the N atoms of the *EnrH^+^* and the O atoms of the water molecules as donor atoms, and the Cl^-^ anions and the O atoms of the carboxyl groups as acceptors. The O1W—H1D···Cl1, O1W—H1E···Cl2, O3W—H3D···Cl1 and O3A—H3E···Cl2 hydrogen bonds form an *R*_4_^2^(8) motif (Etter *et al.*, 1990), while the N3A—H3F···Cl2, O4W—H4D···Cl2 and O4W—O4E···O3A hydrogen bonds form a *C*_3_^2^(17) motif running along the crystallographic *c* axis. Finally, *A* and *B* molecules form a π–π interaction between *Cg*4···*Cg*9 [3.6726 (13) Å], where *Cg*4 and *Cg*9 are the centroids of the C1A–C6A, C1B–C6B benzene rings, respectively.

### 2. Experimental

The enrofloxacin hydrochloride crystals (Sumano & Gutierrez, 2013) were formed after one month by slow evaporation at room temperature from saturated solution in a mixture of water-ethanol-acetone (3:2:1). Single crystals for X-ray determination were separated by filtration with 0.45 µ*m*-pore membrane and vacuum. Mass spectrum of enrofloxaxin hydrochloride presents two principal signals around to m/*z* 394 and 753 (*M*-), four peaks at m/*z* 394.1313, 395.1336, 396.1285 and 397.1305 (*M*+) corresponding to the deprotonated molecular ion [*M* – H]^-^ with the characteristic isotopic pattern (3:1) that confirms the presence of one chlorine atom.

### 3. Refinement

H atoms of the hydroxy groups and the amine groups were located in a difference map and their positions were refined with bond-length restraints of O—H = 0.86 (2) Å and N—H = 0.92 (2) Å, and with *U*_iso_(H) = 1.5*U*_eq_(O) and 1.2*U*_eq_(N). H atoms attached to C atoms were placed in geometrically idealized positions, and refined as riding on their parent atoms, with C—H distances of 0.95–1.00 Å, and with *U*_iso_(H) = 1.2*U*_eq_(C) or 1.5*U*_eq_(C_methyl_).

### Crystal data

**Table:**

C_19_H_23_FN_3_O_3_^+^·Cl^−^·2H_2_O	*F*(000) = 1824
*M**_r_* = 431.88	*D*_x_ = 1.424 Mg m^−^^3^
Monoclinic, *P*2_1_/*c*	Mo *K*α radiation, λ = 0.71073 Å
Hall symbol: -P 2ybc	Cell parameters from 3358 reflections
*a* = 7.1874 (3) Å	θ = 3.3–29.2°
*b* = 21.1475 (8) Å	µ = 0.24 mm^−^^1^
*c* = 26.5106 (10) Å	*T* = 130 K
β = 91.407 (4)°	Lamina, colourless
*V* = 4028.3 (3) Å^3^	0.47 × 0.14 × 0.04 mm
*Z* = 8	

### Data collection

**Table:**

Agilent Xcalibur (Atlas, Gemini) diffractometer	9291 independent reflections
Graphite monochromator	5799 reflections with *I* > 2σ(*I*)
Detector resolution: 10.4685 pixels mm^-1^	*R*_int_ = 0.044
ω scans	θ_max_ = 29.2°, θ_min_ = 3.3°
Absorption correction: analytical (*CrysAlis RED*; Agilent, 2012)	*h* = −9→9
*T*_min_ = 0.939, *T*_max_ = 0.992	*k* = −26→26
18668 measured reflections	*l* = −36→19

### Refinement

**Table:**

Refinement on *F*^2^	12 restraints
Least-squares matrix: full	Hydrogen site location: mixed
*R*[*F*^2^ > 2σ(*F*^2^)] = 0.058	H atoms treated by a mixture of independent and constrained refinement
*wR*(*F*^2^) = 0.132	*w* = 1/[σ^2^(*F*_o_^2^) + (0.0459*P*)^2^ + 0.4843*P*] where *P* = (*F*_o_^2^ + 2*F*_c_^2^)/3
*S* = 1.02	(Δ/σ)_max_ = 0.001
9291 reflections	Δρ_max_ = 0.39 e Å^−^^3^
561 parameters	Δρ_min_ = −0.29 e Å^−^^3^

### Special details

**Table:**

Geometry. All e.s.d.'s (except the e.s.d. in the dihedral angle between two l.s. planes) are estimated using the full covariance matrix. The cell e.s.d.'s are taken into account individually in the estimation of e.s.d.'s in distances, angles and torsion angles; correlations between e.s.d.'s in cell parameters are only used when they are defined by crystal symmetry. An approximate (isotropic) treatment of cell e.s.d.'s is used for estimating e.s.d.'s involving l.s. planes.

### Fractional atomic coordinates and isotropic or equivalent isotropic displacement parameters (Å^2^)

**Table:**

	*x*	*y*	*z*	*U*_iso_*/*U*_eq_	
O1W	0.0945 (3)	0.55957 (9)	0.30895 (8)	0.0409 (6)	
H1D	0.103 (5)	0.5612 (15)	0.2768 (7)	0.061*	
H1E	0.170 (4)	0.5291 (12)	0.3170 (13)	0.061*	
O2W	0.0169 (3)	0.66979 (9)	0.14791 (8)	0.0337 (5)	
H2D	0.058 (4)	0.6850 (13)	0.1191 (8)	0.051*	
H2E	0.072 (4)	0.6345 (10)	0.1545 (12)	0.051*	
O3W	0.5109 (3)	0.44605 (10)	0.21637 (9)	0.0458 (6)	
H3D	0.410 (4)	0.4656 (15)	0.2045 (13)	0.069*	
H3E	0.502 (5)	0.4386 (16)	0.2490 (7)	0.069*	
O4W	0.4734 (3)	0.69206 (9)	0.63793 (8)	0.0382 (5)	
H4D	0.524 (4)	0.6547 (10)	0.6419 (13)	0.057*	
H4E	0.541 (4)	0.7093 (14)	0.6137 (10)	0.057*	
Cl1	0.18451 (9)	0.53861 (3)	0.18924 (3)	0.02716 (17)	
Cl2	0.37940 (9)	0.44701 (3)	0.33696 (3)	0.02945 (18)	
C1A	−0.1149 (3)	0.80909 (11)	0.38678 (9)	0.0148 (5)	
C2A	−0.0759 (3)	0.83710 (11)	0.34018 (9)	0.0149 (5)	
H2A	−0.0857	0.8817	0.3365	0.018*	
C3A	−0.0232 (3)	0.80040 (10)	0.29940 (9)	0.0140 (5)	
C4A	−0.0066 (3)	0.73444 (11)	0.30794 (9)	0.0162 (5)	
C5A	−0.0419 (3)	0.70655 (11)	0.35256 (9)	0.0162 (5)	
H5A	−0.0287	0.6621	0.3562	0.019*	
C6A	−0.0981 (3)	0.74348 (11)	0.39352 (9)	0.0149 (5)	
C7A	−0.1400 (3)	0.71392 (11)	0.44110 (9)	0.0167 (5)	
C8A	−0.2098 (3)	0.75514 (11)	0.47927 (9)	0.0170 (5)	
C9A	−0.2239 (3)	0.81822 (11)	0.47027 (9)	0.0176 (5)	
H9A	−0.2718	0.8442	0.4962	0.021*	
C10A	−0.1978 (3)	0.91444 (10)	0.42118 (9)	0.0178 (5)	
H10A	−0.3072	0.9283	0.3999	0.021*	
C11A	−0.1480 (4)	0.95613 (11)	0.46508 (10)	0.0234 (6)	
H11A	−0.0974	0.9358	0.4961	0.028*	
H11B	−0.2266	0.9938	0.4708	0.028*	
C12A	−0.0293 (4)	0.95565 (11)	0.41910 (10)	0.0238 (6)	
H12A	−0.035	0.993	0.3966	0.029*	
H12B	0.0942	0.935	0.4219	0.029*	
C13A	−0.2698 (4)	0.73054 (12)	0.52858 (10)	0.0223 (6)	
C14A	−0.0844 (3)	0.80015 (11)	0.20864 (9)	0.0179 (5)	
H14A	−0.2127	0.8172	0.2084	0.021*	
H14B	−0.0923	0.7536	0.2115	0.021*	
C15A	0.0075 (4)	0.81741 (11)	0.15984 (9)	0.0190 (6)	
H15A	0.1334	0.7985	0.1591	0.023*	
H15B	−0.0665	0.8004	0.1309	0.023*	
C16A	0.1289 (4)	0.91296 (11)	0.20045 (9)	0.0209 (6)	
H16A	0.1368	0.9596	0.1983	0.025*	
H16B	0.2571	0.8958	0.2004	0.025*	
C17A	0.0361 (4)	0.89440 (10)	0.24917 (9)	0.0199 (6)	
H17A	0.1102	0.9107	0.2784	0.024*	
H17B	−0.0895	0.9135	0.2501	0.024*	
C18A	0.1043 (4)	0.90731 (11)	0.10654 (9)	0.0224 (6)	
H18A	0.2404	0.9002	0.1085	0.027*	
H18B	0.0521	0.8803	0.0792	0.027*	
C19A	0.0673 (5)	0.97526 (13)	0.09384 (11)	0.0445 (9)	
H19A	−0.067	0.9832	0.094	0.067*	
H19B	0.1146	0.9846	0.0603	0.067*	
H19C	0.1301	1.0024	0.1189	0.067*	
O1A	−0.1157 (2)	0.65518 (7)	0.44859 (6)	0.0225 (4)	
O2A	−0.2468 (3)	0.66868 (9)	0.53535 (7)	0.0300 (5)	
H2F	−0.190 (4)	0.6560 (13)	0.5082 (9)	0.045*	
O3A	−0.3382 (3)	0.76322 (9)	0.56093 (7)	0.0295 (5)	
F1A	0.0539 (2)	0.69805 (6)	0.26952 (5)	0.0221 (3)	
N1A	−0.1745 (3)	0.84621 (9)	0.42712 (7)	0.0155 (4)	
N2A	0.0212 (3)	0.82566 (9)	0.25263 (7)	0.0162 (4)	
N3A	0.0213 (3)	0.88804 (9)	0.15558 (7)	0.0173 (5)	
H3F	−0.101 (2)	0.9044 (10)	0.1576 (9)	0.021*	
C1B	0.3977 (3)	0.70876 (11)	0.39328 (9)	0.0150 (5)	
C2B	0.4493 (3)	0.67576 (11)	0.34998 (9)	0.0164 (5)	
H2B	0.4524	0.6309	0.3506	0.02*	
C3B	0.4960 (3)	0.70751 (10)	0.30629 (9)	0.0153 (5)	
C4B	0.4858 (3)	0.77417 (11)	0.30757 (9)	0.0168 (5)	
C5B	0.4369 (3)	0.80739 (11)	0.34893 (9)	0.0163 (5)	
H5B	0.4323	0.8523	0.3478	0.02*	
C6B	0.3931 (3)	0.77515 (11)	0.39352 (9)	0.0155 (5)	
C7B	0.3430 (3)	0.80974 (11)	0.43830 (9)	0.0178 (5)	
C8B	0.2841 (3)	0.77203 (12)	0.47991 (9)	0.0187 (5)	
C9B	0.2880 (3)	0.70748 (11)	0.47683 (9)	0.0189 (5)	
H9B	0.2475	0.6839	0.505	0.023*	
C10B	0.3296 (4)	0.60660 (11)	0.43491 (10)	0.0226 (6)	
H10B	0.2198	0.5897	0.4153	0.027*	
C11B	0.3874 (4)	0.56938 (12)	0.48049 (10)	0.0300 (7)	
H11C	0.3132	0.5315	0.4888	0.036*	
H11D	0.4391	0.5928	0.51	0.036*	
C12B	0.5007 (4)	0.56722 (11)	0.43374 (10)	0.0258 (6)	
H12C	0.6222	0.5893	0.4345	0.031*	
H12D	0.4963	0.528	0.4134	0.031*	
C13B	0.2142 (4)	0.80098 (13)	0.52658 (10)	0.0241 (6)	
C14B	0.4501 (4)	0.68711 (11)	0.21643 (9)	0.0187 (5)	
H14C	0.4277	0.733	0.2119	0.022*	
H14D	0.3278	0.666	0.2191	0.022*	
C15B	0.5496 (4)	0.66151 (11)	0.17080 (9)	0.0206 (6)	
H15C	0.4704	0.6672	0.1401	0.025*	
H15D	0.6672	0.685	0.1662	0.025*	
C16B	0.7079 (4)	0.58492 (12)	0.22539 (9)	0.0223 (6)	
H16C	0.8261	0.6084	0.2219	0.027*	
H16D	0.7381	0.5397	0.2304	0.027*	
C17B	0.6058 (4)	0.60954 (11)	0.27052 (9)	0.0205 (6)	
H17C	0.49	0.585	0.2748	0.025*	
H17D	0.6847	0.6043	0.3014	0.025*	
C18B	0.6794 (4)	0.56056 (12)	0.13452 (10)	0.0267 (6)	
H18C	0.8002	0.5812	0.128	0.032*	
H18D	0.7046	0.5158	0.1433	0.032*	
C19B	0.5603 (4)	0.56290 (14)	0.08741 (10)	0.0367 (7)	
H19D	0.4348	0.548	0.0948	0.055*	
H19E	0.6144	0.5357	0.0617	0.055*	
H19F	0.5539	0.6065	0.075	0.055*	
O1B	0.3504 (2)	0.86958 (8)	0.44026 (7)	0.0243 (4)	
O2B	0.2142 (3)	0.86369 (9)	0.52735 (7)	0.0320 (5)	
H2G	0.260 (4)	0.8788 (13)	0.4997 (9)	0.048*	
O3B	0.1561 (3)	0.77044 (9)	0.56158 (7)	0.0322 (5)	
F1B	0.5322 (2)	0.80645 (6)	0.26545 (5)	0.0238 (3)	
N1B	0.3457 (3)	0.67547 (9)	0.43647 (7)	0.0167 (4)	
N2B	0.5603 (3)	0.67658 (9)	0.26343 (7)	0.0165 (4)	
N3B	0.5907 (3)	0.59264 (9)	0.17851 (8)	0.0201 (5)	
H3G	0.475 (3)	0.5729 (11)	0.1830 (9)	0.024*	

### Atomic displacement parameters (Å^2^)

**Table:**

	*U*^11^	*U*^22^	*U*^33^	*U*^12^	*U*^13^	*U*^23^
O1W	0.0560 (15)	0.0290 (11)	0.0383 (13)	0.0112 (10)	0.0126 (12)	0.0045 (10)
O2W	0.0407 (13)	0.0304 (11)	0.0307 (12)	0.0032 (10)	0.0125 (10)	0.0072 (9)
O3W	0.0470 (15)	0.0321 (12)	0.0586 (16)	0.0085 (10)	0.0119 (13)	0.0091 (11)
O4W	0.0393 (13)	0.0339 (12)	0.0421 (14)	0.0032 (10)	0.0144 (11)	0.0092 (10)
Cl1	0.0243 (4)	0.0271 (3)	0.0300 (4)	−0.0037 (3)	0.0005 (3)	0.0033 (3)
Cl2	0.0238 (4)	0.0259 (4)	0.0387 (4)	−0.0018 (3)	0.0021 (3)	0.0007 (3)
C1A	0.0108 (12)	0.0205 (12)	0.0131 (12)	0.0012 (10)	−0.0001 (10)	−0.0016 (10)
C2A	0.0146 (12)	0.0140 (11)	0.0161 (13)	−0.0014 (10)	−0.0002 (10)	0.0013 (10)
C3A	0.0082 (12)	0.0175 (12)	0.0163 (13)	−0.0001 (10)	0.0000 (10)	−0.0010 (10)
C4A	0.0130 (12)	0.0201 (12)	0.0154 (13)	0.0028 (10)	−0.0007 (10)	−0.0059 (10)
C5A	0.0132 (13)	0.0163 (12)	0.0190 (13)	0.0017 (10)	−0.0023 (10)	0.0001 (10)
C6A	0.0112 (12)	0.0185 (12)	0.0148 (13)	−0.0010 (10)	−0.0024 (10)	−0.0013 (10)
C7A	0.0116 (12)	0.0205 (13)	0.0177 (13)	−0.0019 (10)	−0.0029 (10)	0.0006 (10)
C8A	0.0135 (12)	0.0245 (13)	0.0130 (13)	−0.0023 (11)	−0.0015 (10)	0.0017 (10)
C9A	0.0121 (12)	0.0281 (14)	0.0126 (12)	0.0001 (11)	0.0001 (10)	−0.0021 (10)
C10A	0.0204 (14)	0.0159 (12)	0.0170 (13)	0.0031 (10)	−0.0012 (11)	−0.0014 (10)
C11A	0.0324 (16)	0.0173 (13)	0.0204 (14)	0.0013 (11)	−0.0013 (12)	−0.0047 (11)
C12A	0.0262 (15)	0.0201 (13)	0.0252 (15)	−0.0025 (11)	0.0001 (12)	−0.0002 (11)
C13A	0.0211 (14)	0.0289 (15)	0.0166 (14)	−0.0043 (12)	−0.0037 (12)	0.0050 (11)
C14A	0.0185 (14)	0.0192 (13)	0.0160 (13)	−0.0027 (10)	−0.0005 (11)	0.0005 (10)
C15A	0.0246 (14)	0.0197 (13)	0.0128 (13)	0.0014 (11)	0.0016 (11)	−0.0024 (10)
C16A	0.0243 (15)	0.0192 (13)	0.0193 (14)	−0.0073 (11)	0.0029 (12)	−0.0005 (11)
C17A	0.0267 (15)	0.0154 (12)	0.0178 (14)	−0.0052 (11)	0.0029 (12)	−0.0018 (10)
C18A	0.0240 (15)	0.0283 (14)	0.0151 (13)	−0.0003 (12)	0.0052 (11)	0.0019 (11)
C19A	0.066 (2)	0.0362 (17)	0.0326 (18)	0.0047 (16)	0.0245 (17)	0.0125 (14)
O1A	0.0251 (10)	0.0211 (9)	0.0214 (10)	0.0008 (8)	0.0015 (8)	0.0038 (7)
O2A	0.0404 (13)	0.0302 (11)	0.0197 (10)	−0.0025 (9)	0.0057 (9)	0.0072 (8)
O3A	0.0358 (12)	0.0375 (11)	0.0155 (10)	−0.0019 (9)	0.0080 (9)	0.0012 (8)
F1A	0.0310 (9)	0.0198 (7)	0.0158 (8)	0.0054 (6)	0.0047 (7)	−0.0039 (6)
N1A	0.0145 (10)	0.0183 (10)	0.0137 (11)	0.0002 (8)	0.0003 (9)	−0.0018 (8)
N2A	0.0182 (11)	0.0179 (10)	0.0126 (10)	−0.0016 (9)	0.0023 (9)	−0.0013 (8)
N3A	0.0178 (11)	0.0187 (11)	0.0155 (11)	0.0013 (9)	0.0034 (9)	−0.0010 (8)
C1B	0.0116 (12)	0.0191 (12)	0.0142 (13)	0.0001 (10)	−0.0008 (10)	0.0008 (10)
C2B	0.0154 (13)	0.0144 (12)	0.0195 (13)	0.0013 (10)	0.0002 (11)	−0.0006 (10)
C3B	0.0126 (12)	0.0168 (12)	0.0166 (13)	−0.0010 (10)	−0.0008 (10)	−0.0021 (10)
C4B	0.0150 (13)	0.0204 (12)	0.0151 (13)	−0.0023 (10)	0.0023 (11)	0.0027 (10)
C5B	0.0147 (13)	0.0144 (12)	0.0199 (13)	−0.0007 (10)	0.0018 (11)	−0.0012 (10)
C6B	0.0100 (12)	0.0207 (12)	0.0158 (13)	−0.0005 (10)	−0.0014 (10)	−0.0038 (10)
C7B	0.0090 (12)	0.0228 (13)	0.0214 (14)	0.0016 (10)	−0.0030 (10)	−0.0045 (11)
C8B	0.0132 (13)	0.0280 (14)	0.0148 (13)	0.0009 (11)	−0.0005 (10)	−0.0027 (11)
C9B	0.0145 (13)	0.0295 (14)	0.0128 (13)	0.0005 (11)	0.0008 (10)	0.0004 (11)
C10B	0.0253 (15)	0.0200 (13)	0.0227 (15)	−0.0016 (11)	0.0043 (12)	0.0034 (11)
C11B	0.0393 (18)	0.0242 (14)	0.0268 (16)	0.0014 (13)	0.0062 (14)	0.0076 (12)
C12B	0.0309 (16)	0.0209 (13)	0.0259 (15)	0.0027 (12)	0.0033 (13)	0.0010 (11)
C13B	0.0182 (14)	0.0351 (16)	0.0188 (14)	0.0046 (12)	−0.0038 (12)	−0.0057 (12)
C14B	0.0195 (14)	0.0216 (13)	0.0150 (13)	0.0003 (11)	−0.0014 (11)	−0.0024 (10)
C15B	0.0232 (14)	0.0235 (13)	0.0152 (13)	−0.0014 (11)	0.0018 (11)	−0.0013 (11)
C16B	0.0243 (15)	0.0219 (13)	0.0207 (14)	0.0031 (11)	0.0007 (12)	−0.0047 (11)
C17B	0.0218 (14)	0.0217 (13)	0.0182 (14)	0.0021 (11)	0.0029 (11)	−0.0020 (11)
C18B	0.0291 (16)	0.0302 (15)	0.0212 (14)	−0.0004 (12)	0.0079 (12)	−0.0086 (12)
C19B	0.0393 (19)	0.0488 (19)	0.0222 (16)	−0.0016 (15)	0.0030 (14)	−0.0124 (13)
O1B	0.0275 (11)	0.0197 (9)	0.0257 (10)	0.0005 (8)	0.0028 (8)	−0.0066 (8)
O2B	0.0401 (13)	0.0320 (11)	0.0242 (11)	0.0035 (9)	0.0052 (10)	−0.0112 (9)
O3B	0.0371 (12)	0.0428 (12)	0.0171 (10)	0.0044 (9)	0.0072 (9)	−0.0031 (9)
F1B	0.0338 (9)	0.0209 (7)	0.0169 (8)	−0.0037 (6)	0.0085 (7)	0.0028 (6)
N1B	0.0153 (11)	0.0195 (11)	0.0154 (11)	0.0006 (9)	0.0014 (9)	0.0032 (9)
N2B	0.0176 (11)	0.0177 (10)	0.0145 (11)	0.0025 (9)	0.0034 (9)	−0.0012 (8)
N3B	0.0196 (12)	0.0206 (11)	0.0204 (12)	−0.0032 (9)	0.0061 (10)	−0.0056 (9)

### Geometric parameters (Å, º)

**Table:**

O1W—H1D	0.858 (18)	C19A—H19B	0.98
O1W—H1E	0.866 (18)	C19A—H19C	0.98
O2W—H2D	0.886 (17)	O2A—H2F	0.876 (17)
O2W—H2E	0.859 (17)	N3A—H3F	0.946 (16)
O3W—H3D	0.888 (18)	C1B—C2B	1.401 (3)
O3W—H3E	0.883 (18)	C1B—N1B	1.403 (3)
O4W—H4D	0.875 (17)	C1B—C6B	1.404 (3)
O4W—H4E	0.895 (18)	C2B—C3B	1.387 (3)
C1A—N1A	1.402 (3)	C2B—H2B	0.95
C1A—C6A	1.404 (3)	C3B—N2B	1.399 (3)
C1A—C2A	1.404 (3)	C3B—C4B	1.412 (3)
C2A—C3A	1.391 (3)	C4B—C5B	1.356 (3)
C2A—H2A	0.95	C4B—F1B	1.358 (3)
C3A—N2A	1.394 (3)	C5B—C6B	1.407 (3)
C3A—C4A	1.418 (3)	C5B—H5B	0.95
C4A—C5A	1.352 (3)	C6B—C7B	1.447 (3)
C4A—F1A	1.357 (3)	C7B—O1B	1.268 (3)
C5A—C6A	1.405 (3)	C7B—C8B	1.433 (3)
C5A—H5A	0.95	C8B—C9B	1.368 (3)
C6A—C7A	1.446 (3)	C8B—C13B	1.479 (3)
C7A—O1A	1.269 (3)	C9B—N1B	1.340 (3)
C7A—C8A	1.436 (3)	C9B—H9B	0.95
C8A—C9A	1.358 (3)	C10B—N1B	1.462 (3)
C8A—C13A	1.481 (3)	C10B—C12B	1.486 (4)
C9A—N1A	1.344 (3)	C10B—C11B	1.492 (3)
C9A—H9A	0.95	C10B—H10B	1
C10A—N1A	1.461 (3)	C11B—C12B	1.500 (3)
C10A—C12A	1.494 (3)	C11B—H11C	0.99
C10A—C11A	1.496 (3)	C11B—H11D	0.99
C10A—H10A	1	C12B—H12C	0.99
C11A—C12A	1.505 (3)	C12B—H12D	0.99
C11A—H11A	0.99	C13B—O3B	1.213 (3)
C11A—H11B	0.99	C13B—O2B	1.326 (3)
C12A—H12A	0.99	C14B—N2B	1.477 (3)
C12A—H12B	0.99	C14B—C15B	1.520 (3)
C13A—O3A	1.215 (3)	C14B—H14C	0.99
C13A—O2A	1.330 (3)	C14B—H14D	0.99
C14A—N2A	1.478 (3)	C15B—N3B	1.499 (3)
C14A—C15A	1.512 (3)	C15B—H15C	0.99
C14A—H14A	0.99	C15B—H15D	0.99
C14A—H14B	0.99	C16B—N3B	1.493 (3)
C15A—N3A	1.501 (3)	C16B—C17B	1.511 (3)
C15A—H15A	0.99	C16B—H16C	0.99
C15A—H15B	0.99	C16B—H16D	0.99
C16A—N3A	1.499 (3)	C17B—N2B	1.466 (3)
C16A—C17A	1.519 (3)	C17B—H17C	0.99
C16A—H16A	0.99	C17B—H17D	0.99
C16A—H16B	0.99	C18B—C19B	1.498 (4)
C17A—N2A	1.461 (3)	C18B—N3B	1.505 (3)
C17A—H17A	0.99	C18B—H18C	0.99
C17A—H17B	0.99	C18B—H18D	0.99
C18A—C19A	1.498 (4)	C19B—H19D	0.98
C18A—N3A	1.500 (3)	C19B—H19E	0.98
C18A—H18A	0.99	C19B—H19F	0.98
C18A—H18B	0.99	O2B—H2G	0.871 (17)
C19A—H19A	0.98	N3B—H3G	0.942 (16)
			
H1D—O1W—H1E	102 (3)	C18A—N3A—H3F	109.9 (15)
H2D—O2W—H2E	109 (3)	C15A—N3A—H3F	107.2 (14)
H3D—O3W—H3E	110 (4)	C2B—C1B—N1B	120.0 (2)
H4D—O4W—H4E	103 (3)	C2B—C1B—C6B	120.6 (2)
N1A—C1A—C6A	118.9 (2)	N1B—C1B—C6B	119.4 (2)
N1A—C1A—C2A	120.4 (2)	C3B—C2B—C1B	121.2 (2)
C6A—C1A—C2A	120.7 (2)	C3B—C2B—H2B	119.4
C3A—C2A—C1A	120.8 (2)	C1B—C2B—H2B	119.4
C3A—C2A—H2A	119.6	C2B—C3B—N2B	122.9 (2)
C1A—C2A—H2A	119.6	C2B—C3B—C4B	116.7 (2)
C2A—C3A—N2A	123.4 (2)	N2B—C3B—C4B	120.3 (2)
C2A—C3A—C4A	116.6 (2)	C5B—C4B—F1B	118.6 (2)
N2A—C3A—C4A	119.9 (2)	C5B—C4B—C3B	123.5 (2)
C5A—C4A—F1A	118.7 (2)	F1B—C4B—C3B	117.9 (2)
C5A—C4A—C3A	123.5 (2)	C4B—C5B—C6B	119.8 (2)
F1A—C4A—C3A	117.8 (2)	C4B—C5B—H5B	120.1
C4A—C5A—C6A	119.8 (2)	C6B—C5B—H5B	120.1
C4A—C5A—H5A	120.1	C1B—C6B—C5B	118.3 (2)
C6A—C5A—H5A	120.1	C1B—C6B—C7B	121.0 (2)
C1A—C6A—C5A	118.5 (2)	C5B—C6B—C7B	120.6 (2)
C1A—C6A—C7A	121.3 (2)	O1B—C7B—C8B	122.4 (2)
C5A—C6A—C7A	120.2 (2)	O1B—C7B—C6B	121.8 (2)
O1A—C7A—C8A	122.2 (2)	C8B—C7B—C6B	115.7 (2)
O1A—C7A—C6A	121.9 (2)	C9B—C8B—C7B	120.1 (2)
C8A—C7A—C6A	115.9 (2)	C9B—C8B—C13B	118.1 (2)
C9A—C8A—C7A	119.9 (2)	C7B—C8B—C13B	121.8 (2)
C9A—C8A—C13A	118.5 (2)	N1B—C9B—C8B	124.0 (2)
C7A—C8A—C13A	121.6 (2)	N1B—C9B—H9B	118
N1A—C9A—C8A	124.2 (2)	C8B—C9B—H9B	118
N1A—C9A—H9A	117.9	N1B—C10B—C12B	119.6 (2)
C8A—C9A—H9A	117.9	N1B—C10B—C11B	118.8 (2)
N1A—C10A—C12A	119.3 (2)	C12B—C10B—C11B	60.49 (17)
N1A—C10A—C11A	118.3 (2)	N1B—C10B—H10B	115.6
C12A—C10A—C11A	60.44 (16)	C12B—C10B—H10B	115.6
N1A—C10A—H10A	115.8	C11B—C10B—H10B	115.6
C12A—C10A—H10A	115.8	C10B—C11B—C12B	59.53 (17)
C11A—C10A—H10A	115.8	C10B—C11B—H11C	117.8
C10A—C11A—C12A	59.70 (16)	C12B—C11B—H11C	117.8
C10A—C11A—H11A	117.8	C10B—C11B—H11D	117.8
C12A—C11A—H11A	117.8	C12B—C11B—H11D	117.8
C10A—C11A—H11B	117.8	H11C—C11B—H11D	115
C12A—C11A—H11B	117.8	C10B—C12B—C11B	59.98 (17)
H11A—C11A—H11B	114.9	C10B—C12B—H12C	117.8
C10A—C12A—C11A	59.86 (16)	C11B—C12B—H12C	117.8
C10A—C12A—H12A	117.8	C10B—C12B—H12D	117.8
C11A—C12A—H12A	117.8	C11B—C12B—H12D	117.8
C10A—C12A—H12B	117.8	H12C—C12B—H12D	114.9
C11A—C12A—H12B	117.8	O3B—C13B—O2B	121.4 (2)
H12A—C12A—H12B	114.9	O3B—C13B—C8B	123.4 (2)
O3A—C13A—O2A	121.0 (2)	O2B—C13B—C8B	115.3 (2)
O3A—C13A—C8A	123.7 (2)	N2B—C14B—C15B	111.4 (2)
O2A—C13A—C8A	115.3 (2)	N2B—C14B—H14C	109.4
N2A—C14A—C15A	111.13 (19)	C15B—C14B—H14C	109.4
N2A—C14A—H14A	109.4	N2B—C14B—H14D	109.4
C15A—C14A—H14A	109.4	C15B—C14B—H14D	109.4
N2A—C14A—H14B	109.4	H14C—C14B—H14D	108
C15A—C14A—H14B	109.4	N3B—C15B—C14B	109.41 (19)
H14A—C14A—H14B	108	N3B—C15B—H15C	109.8
N3A—C15A—C14A	109.61 (18)	C14B—C15B—H15C	109.8
N3A—C15A—H15A	109.7	N3B—C15B—H15D	109.8
C14A—C15A—H15A	109.7	C14B—C15B—H15D	109.8
N3A—C15A—H15B	109.7	H15C—C15B—H15D	108.2
C14A—C15A—H15B	109.7	N3B—C16B—C17B	110.3 (2)
H15A—C15A—H15B	108.2	N3B—C16B—H16C	109.6
N3A—C16A—C17A	110.8 (2)	C17B—C16B—H16C	109.6
N3A—C16A—H16A	109.5	N3B—C16B—H16D	109.6
C17A—C16A—H16A	109.5	C17B—C16B—H16D	109.6
N3A—C16A—H16B	109.5	H16C—C16B—H16D	108.1
C17A—C16A—H16B	109.5	N2B—C17B—C16B	110.04 (19)
H16A—C16A—H16B	108.1	N2B—C17B—H17C	109.7
N2A—C17A—C16A	110.18 (18)	C16B—C17B—H17C	109.7
N2A—C17A—H17A	109.6	N2B—C17B—H17D	109.7
C16A—C17A—H17A	109.6	C16B—C17B—H17D	109.7
N2A—C17A—H17B	109.6	H17C—C17B—H17D	108.2
C16A—C17A—H17B	109.6	C19B—C18B—N3B	112.8 (2)
H17A—C17A—H17B	108.1	C19B—C18B—H18C	109
C19A—C18A—N3A	112.5 (2)	N3B—C18B—H18C	109
C19A—C18A—H18A	109.1	C19B—C18B—H18D	109
N3A—C18A—H18A	109.1	N3B—C18B—H18D	109
C19A—C18A—H18B	109.1	H18C—C18B—H18D	107.8
N3A—C18A—H18B	109.1	C18B—C19B—H19D	109.5
H18A—C18A—H18B	107.8	C18B—C19B—H19E	109.5
C18A—C19A—H19A	109.5	H19D—C19B—H19E	109.5
C18A—C19A—H19B	109.5	C18B—C19B—H19F	109.5
H19A—C19A—H19B	109.5	H19D—C19B—H19F	109.5
C18A—C19A—H19C	109.5	H19E—C19B—H19F	109.5
H19A—C19A—H19C	109.5	C13B—O2B—H2G	111 (2)
H19B—C19A—H19C	109.5	C9B—N1B—C1B	119.5 (2)
C13A—O2A—H2F	104.4 (19)	C9B—N1B—C10B	120.02 (19)
C9A—N1A—C1A	119.7 (2)	C1B—N1B—C10B	119.96 (18)
C9A—N1A—C10A	119.66 (19)	C3B—N2B—C17B	115.15 (18)
C1A—N1A—C10A	120.48 (19)	C3B—N2B—C14B	115.70 (19)
C3A—N2A—C17A	117.13 (18)	C17B—N2B—C14B	111.56 (18)
C3A—N2A—C14A	115.95 (19)	C16B—N3B—C15B	108.96 (18)
C17A—N2A—C14A	110.55 (18)	C16B—N3B—C18B	110.78 (19)
C16A—N3A—C18A	112.58 (19)	C15B—N3B—C18B	114.72 (19)
C16A—N3A—C15A	108.90 (18)	C16B—N3B—H3G	109.2 (16)
C18A—N3A—C15A	111.34 (17)	C15B—N3B—H3G	106.0 (15)
C16A—N3A—H3F	106.7 (15)	C18B—N3B—H3G	106.9 (16)
			
N1A—C1A—C2A—C3A	−177.7 (2)	N1B—C1B—C2B—C3B	178.0 (2)
C6A—C1A—C2A—C3A	1.2 (3)	C6B—C1B—C2B—C3B	−0.3 (4)
C1A—C2A—C3A—N2A	−178.5 (2)	C1B—C2B—C3B—N2B	175.5 (2)
C1A—C2A—C3A—C4A	−1.6 (3)	C1B—C2B—C3B—C4B	−1.0 (3)
C2A—C3A—C4A—C5A	1.1 (4)	C2B—C3B—C4B—C5B	1.2 (4)
N2A—C3A—C4A—C5A	178.1 (2)	N2B—C3B—C4B—C5B	−175.4 (2)
C2A—C3A—C4A—F1A	−176.7 (2)	C2B—C3B—C4B—F1B	179.4 (2)
N2A—C3A—C4A—F1A	0.3 (3)	N2B—C3B—C4B—F1B	2.8 (3)
F1A—C4A—C5A—C6A	177.7 (2)	F1B—C4B—C5B—C6B	−178.3 (2)
C3A—C4A—C5A—C6A	−0.1 (4)	C3B—C4B—C5B—C6B	−0.1 (4)
N1A—C1A—C6A—C5A	178.7 (2)	C2B—C1B—C6B—C5B	1.5 (3)
C2A—C1A—C6A—C5A	−0.1 (3)	N1B—C1B—C6B—C5B	−176.9 (2)
N1A—C1A—C6A—C7A	−0.4 (3)	C2B—C1B—C6B—C7B	−179.0 (2)
C2A—C1A—C6A—C7A	−179.2 (2)	N1B—C1B—C6B—C7B	2.6 (3)
C4A—C5A—C6A—C1A	−0.4 (3)	C4B—C5B—C6B—C1B	−1.3 (4)
C4A—C5A—C6A—C7A	178.7 (2)	C4B—C5B—C6B—C7B	179.2 (2)
C1A—C6A—C7A—O1A	−176.1 (2)	C1B—C6B—C7B—O1B	175.1 (2)
C5A—C6A—C7A—O1A	4.9 (4)	C5B—C6B—C7B—O1B	−5.4 (4)
C1A—C6A—C7A—C8A	3.7 (3)	C1B—C6B—C7B—C8B	−5.2 (3)
C5A—C6A—C7A—C8A	−175.4 (2)	C5B—C6B—C7B—C8B	174.3 (2)
O1A—C7A—C8A—C9A	176.5 (2)	O1B—C7B—C8B—C9B	−176.5 (2)
C6A—C7A—C8A—C9A	−3.3 (3)	C6B—C7B—C8B—C9B	3.8 (3)
O1A—C7A—C8A—C13A	−4.2 (4)	O1B—C7B—C8B—C13B	4.8 (4)
C6A—C7A—C8A—C13A	176.0 (2)	C6B—C7B—C8B—C13B	−174.9 (2)
C7A—C8A—C9A—N1A	−0.6 (4)	C7B—C8B—C9B—N1B	0.3 (4)
C13A—C8A—C9A—N1A	−179.9 (2)	C13B—C8B—C9B—N1B	179.1 (2)
N1A—C10A—C11A—C12A	109.5 (2)	N1B—C10B—C11B—C12B	−109.7 (3)
N1A—C10A—C12A—C11A	−107.8 (2)	N1B—C10B—C12B—C11B	108.4 (3)
C9A—C8A—C13A—O3A	3.1 (4)	C9B—C8B—C13B—O3B	−1.9 (4)
C7A—C8A—C13A—O3A	−176.2 (2)	C7B—C8B—C13B—O3B	176.8 (2)
C9A—C8A—C13A—O2A	−178.0 (2)	C9B—C8B—C13B—O2B	179.8 (2)
C7A—C8A—C13A—O2A	2.7 (3)	C7B—C8B—C13B—O2B	−1.4 (4)
N2A—C14A—C15A—N3A	−58.5 (3)	N2B—C14B—C15B—N3B	56.7 (3)
N3A—C16A—C17A—N2A	58.4 (3)	N3B—C16B—C17B—N2B	−59.2 (3)
C8A—C9A—N1A—C1A	4.2 (4)	C8B—C9B—N1B—C1B	−3.1 (4)
C8A—C9A—N1A—C10A	179.2 (2)	C8B—C9B—N1B—C10B	−174.6 (2)
C6A—C1A—N1A—C9A	−3.6 (3)	C2B—C1B—N1B—C9B	−176.8 (2)
C2A—C1A—N1A—C9A	175.3 (2)	C6B—C1B—N1B—C9B	1.6 (3)
C6A—C1A—N1A—C10A	−178.6 (2)	C2B—C1B—N1B—C10B	−5.3 (3)
C2A—C1A—N1A—C10A	0.3 (3)	C6B—C1B—N1B—C10B	173.1 (2)
C12A—C10A—N1A—C9A	111.5 (3)	C12B—C10B—N1B—C9B	−115.5 (3)
C11A—C10A—N1A—C9A	41.4 (3)	C11B—C10B—N1B—C9B	−45.0 (3)
C12A—C10A—N1A—C1A	−73.5 (3)	C12B—C10B—N1B—C1B	73.0 (3)
C11A—C10A—N1A—C1A	−143.6 (2)	C11B—C10B—N1B—C1B	143.5 (2)
C2A—C3A—N2A—C17A	8.4 (3)	C2B—C3B—N2B—C17B	−10.1 (3)
C4A—C3A—N2A—C17A	−168.4 (2)	C4B—C3B—N2B—C17B	166.4 (2)
C2A—C3A—N2A—C14A	−125.1 (2)	C2B—C3B—N2B—C14B	122.5 (2)
C4A—C3A—N2A—C14A	58.1 (3)	C4B—C3B—N2B—C14B	−61.1 (3)
C16A—C17A—N2A—C3A	166.5 (2)	C16B—C17B—N2B—C3B	−168.9 (2)
C16A—C17A—N2A—C14A	−57.7 (3)	C16B—C17B—N2B—C14B	56.6 (3)
C15A—C14A—N2A—C3A	−165.0 (2)	C15B—C14B—N2B—C3B	169.8 (2)
C15A—C14A—N2A—C17A	58.6 (2)	C15B—C14B—N2B—C17B	−56.0 (2)
C17A—C16A—N3A—C18A	177.90 (19)	C17B—C16B—N3B—C15B	60.5 (2)
C17A—C16A—N3A—C15A	−58.1 (2)	C17B—C16B—N3B—C18B	−172.4 (2)
C19A—C18A—N3A—C16A	−75.0 (3)	C14B—C15B—N3B—C16B	−58.6 (3)
C19A—C18A—N3A—C15A	162.4 (2)	C14B—C15B—N3B—C18B	176.6 (2)
C14A—C15A—N3A—C16A	57.8 (2)	C19B—C18B—N3B—C16B	175.8 (2)
C14A—C15A—N3A—C18A	−177.5 (2)	C19B—C18B—N3B—C15B	−60.4 (3)

### Hydrogen-bond geometry (Å, º)

**Table:**

*D*—H···*A*	*D*—H	H···*A*	*D*···*A*	*D*—H···*A*
O3*W*—H3*D*···Cl1	0.888 (18)	2.27 (2)	3.126 (2)	163 (3)
O2*W*—H2*E*···Cl1	0.859 (17)	2.363 (19)	3.207 (2)	167 (3)
O4*W*—H4*E*···O3*A*^i^	0.895 (18)	2.015 (19)	2.899 (3)	169 (3)
O3*W*—H3*E*···Cl2	0.883 (18)	2.52 (2)	3.356 (3)	158 (3)
O1*W*—H1*E*···Cl2	0.866 (18)	2.350 (18)	3.215 (2)	179 (3)
O4*W*—H4*D*···Cl2^ii^	0.875 (17)	2.325 (19)	3.190 (2)	170 (3)
O1*W*—H1*D*···Cl1	0.858 (18)	2.45 (2)	3.285 (2)	163 (3)
O2*W*—H2*D*···O3*B*^iii^	0.886 (17)	1.940 (18)	2.819 (3)	172 (3)
O2*A*—H2*F*···O1*A*	0.876 (17)	1.68 (2)	2.523 (2)	160 (3)
O2*B*—H2*G*···O1*B*	0.871 (17)	1.73 (2)	2.532 (3)	152 (3)
N3*B*—H3*G*···Cl1	0.942 (16)	2.219 (17)	3.154 (2)	172 (2)
N3*A*—H3*F*···Cl2^iv^	0.946 (16)	2.204 (17)	3.149 (2)	177 (2)

Symmetry codes: (i) *x*+1, *y*, *z*; (ii) −*x*+1, −*y*+1, −*z*+1; (iii) *x*, −*y*+3/2, *z*−1/2; (iv) −*x*, *y*+1/2, −*z*+1/2.
